# On the Use of Nitrate of Silver in Lacerated Wounds

**Published:** 1850-02

**Authors:** John Higginbottom

**Affiliations:** Nottingham


### SURGERY.

*On the use of Nitrate of Silver in Lacerated Wounds.* By John Higginbottom, Esq., F. R. C. S., Nottingham.

*Lacerated Wounds of the Face.*—Case 1,—Miss R——, aged twenty. On a very windy day, a piece of slate was blown from the roof of a house, which fell upon her forehead, and inflicted a wound of five inches in length, commencing on the forehead, above the right eye, passing obliquely across the nose, and terminating on the left cheek, leaving a large open wound, quite disfiguring the face. The wounded parts, after being well cleansed from extraneous matter, were neatly closed by the interrupted suture; the nitrate of silver was then applied along the edges of the wound, on the line of the wound, and also on the surrounding skin. Afterwards, strips of adhesive plaster were applied, without any other covering.

The wound healed by the first intention, and required no further application. It is now several years since the accident. A common observer standing at a short distance, cannot see the mark of the union, for no disagreeable mark or cicatrix remains.

Great advantages are derived from healing lacerated wounds on the face by the aid of the nitrate of silver.

1. It prevents irritation arising from the irregular edges of a lacerated wound, and it induces adhesive inflammation as readily as in an incised wound.

2. The inflammation, swelling, and irritative fever, consequent on lacerated wounds, are in a great measure prevented, and there being no ulcerative process, there is no loss of substance, so that unsightly scars and raised cicatrices are obviated.

Case 2. A boy was standing on a wall, supporting himself against some iron palisades fixed in the wall. Not having a firm footing, his feet slipped, and he threw his head forward, and his cheek came in contact with a sharp iron spike at the top of one of the palisades: it pierced through the lower part of the cheek, fractured the malar bone, and perforated the upper part of the cheek, so as to cause two lacerated wounds, two inches in length, one above the fractured bone, and the other below.

After the parts had been washed, the wound was closed by two interrupted sutures; the nitrate of silver was applied to the edges of the wound, to destroy their irritability, and an inch upon the surrounding skin; also over the surface of the small line of wound that was left exposed; but not within the wound. Adhesive strips were applied to support the part; no bandage was put over it.

In four days the wounds had healed by the first intention, except a small opening in the lower wound. The ligatures were removed on the third day, and the slight orifices made by the removal of the ligatures were touched with the nitrate of silver, to prevent inflammation or ulceration.

It was observed that a clear fluid flowed from the small opening that remained in the lower wound, which was found to arise from a wound of the salivary duct.

After three applications of the nitrate of silver, to form an adherent eschar, this small opening was closed; and in three weeks there was no further discharge of saliva, the wound being healed,

The use of the nitrate of silver, in parotid fistula, I had previously practised, in a case of seventeen years' duration, published in the *London and Physical Journal* for January, 1830. In this case there were two orifices—one was healed by the nitrate of silver; the other, with the concentrated sulphuric acid.

*Lacerated Perinæum.*—Mrs. H——, on her first labour, drew herself suddenly away from any assistance at the moment of the birth of the head of the child; and in consequence the perinæum was lacerated just above the right side of the median line, entirely through the sphincter ani.

The parts were directly united by the interrupted suture in two places, and the nitrate of silver applied along the skin on each side, close to the line of the wound, and on the line of the wound, and left without any other dressing.

No swelling or inflammation followed to require any other treatment.

On the expiration of the second day, a dose of castor oil was given, which had the desired effect of opening the bowels without disturbing the parts.

On the third day, the sutures were removed, and the ligature marks touched with nitrate of silver to prevent ulceration. The wound united by the first intention; there was no swelling; and it is worthy of observation, that the eschar surrounding the laceration made by the nitrate of silver had the power of fixing the parts as if adhesive plaster had been applied.

No further treatment was required, and the patient experienced no future inconvenience. It is now thirteen years since this case occurred, and the patient has borne nine children, but there has been no other laceration.

On examining the part lately, the mark of the wound is scarcely perceptible, except that one point is a little raised at the anterior edge of the perinæum.

If another case should occur, I should use the catheter to draw off the urine for the first three or four days, although in this case no injury was sustained by its neglect.—*London Lancet.*

